# Supplementary material for: The causal relationship between the human gut microbiota and pyogenic arthritis: a Mendelian randomization study
Source: Front Cell Infect Microbiol. 2024 Nov 26;14:1452480. doi: 10.3389/fcimb.2024.1452480 (PMC11629706; doi:10.3389/fcimb.2024.1452480)
Supplement: Supplementary file 1 [file DataSheet1.zip › mendelian test/ebi-a-GCST90027786.csv_leave_one_out.pdf]

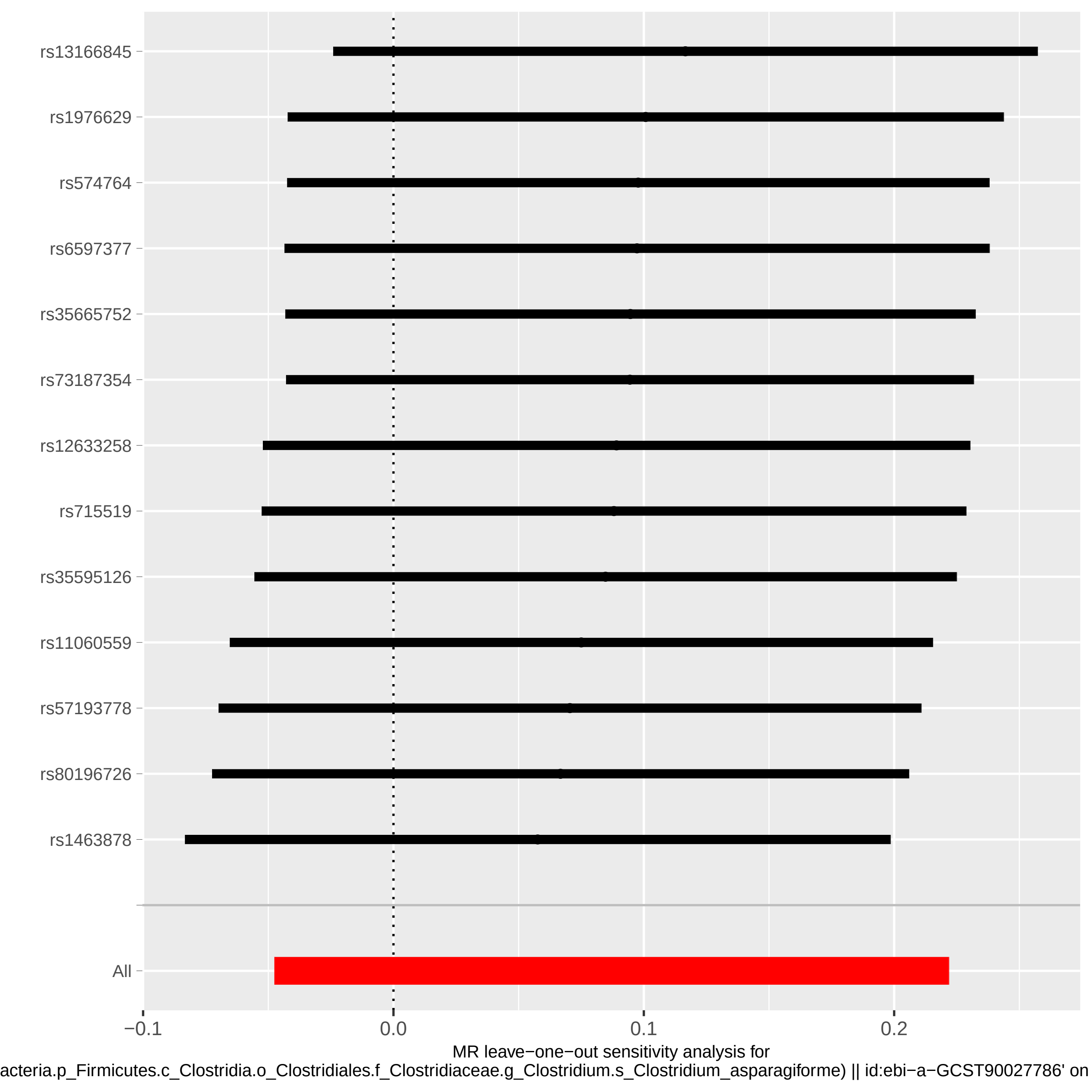

rs13166845
rs1976629
rs574764
rs6597377
rs35665752
rs73187354
rs12633258
rs715519
rs35595126
rs11060559
rs57193778
rs80196726
rs1463878
All
−0.1
0.0
0.1
0.2
MR leave−one−out sensitivity analysis for
acteria.p_Firmicutes.c_Clostridia.o_Clostridiales.f_Clostridiaceae.g_Clostridium.s_Clostridium_asparagiforme) || id:ebi−a−GCST90027786' on
